# Dissemination of definitions and concepts of allergic and hypersensitivity conditions

**DOI:** 10.1186/s40413-016-0115-2

**Published:** 2016-08-09

**Authors:** Luciana Kase Tanno, Moises A. Calderon, Helen E. Smith, Mario Sanchez-Borges, Aziz Sheikh, Pascal Demoly, on behalf of Joint Allergy Academies

**Affiliations:** 1 Hospital Sírio Libanês, São Paulo, Brazil; 2 Division of Allergy, Department of Pulmonology, Hôpital Arnaud de Villeneuve, University Hospital of Montpellier, Montpellier, France; 3 Sorbonne Universités, UPMC Paris 06, UMR-S 1136, IPLESP, Equipe EPAR, 75013 Paris, France; 4 Section of Allergy and Clinical Immunology, Imperial College London, National Heart and Lung Institute, Royal Brompton Hospital, London, UK; 5 Division of Primary Care and Public Health, Brighton and Sussex Medical School, Brighton, UK; 6 Allergy and Clinical Immunology Department, Centro Medico Docente La Trinidad, Caracas, Venezuela; 7 Asthma UK Centre for Applied Research, Usher Institute of Population Health Sciences and Informatics, The University of Edinburgh, Edinburgh, UK

**Keywords:** Allergy, Hypersensitivity, Allergic conditions, Hypersensitivity conditions, Sensitization

## Abstract

**Background:**

Allergy and hypersensitivity can affect people of any age and manifest with problems in a range of organ systems. Moreover, they can have a significant impact on the quality of life of patients and their families. Although once rare, there is presently an epidemic of allergic disorders with associated considerable societal consequences.

Our understanding of the pathophysiology of these disorders has changed substantially over the last 20 years. In the light of these developments, the Joint Allergy Academies have made concerted efforts to ensure that these are reflected in the current definitions and concepts used in clinical allergy and to ensure these are reflected in the forthcoming International Classification of Diseases-11 (ICD-11).

**Objective:**

In this review, we seek to provide an update on the current definitions and concepts in relation to allergic disorders.

**Results:**

Once the new section has been built in the ICD-11 to address allergic and hypersensitivity conditions, we have been moving actions to try to support awareness by disseminating updated concepts in the field. Aligned with the ICD and the WAO philosophy of being global, this document presents fundamental and broad allergy concepts to strengthen the understanding by different health professionals worldwide, besides to support the formation of in training students.

**Conclusion:**

This current review intends to be accepted and used universally by all health professionals involved in diseases’ classification and coding and, therefore, contribute to improve care and outcomes in this increasing sub-section of the world’s population.

## Background

### Allergy and hypersensitivity: stating the problems

#### The importance of disease classification

**Fig. 1:**
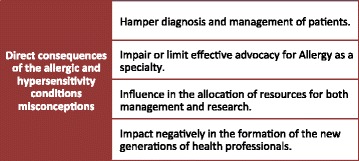
Impact of the allergic and hypersensitivity conditions misconceptions

Many patients have chronic conditions or episodes of illness in which allergy triggers may be implicated. These conditions and episodes can be assigned different classifications and terminologies by different healthcare professionals. Some of these labels are misrepresentative labels leading to misconceptions. These varied working definitions have hampered our real understanding of these conditions. Inaccurate disease classification can lead to suboptimal patient care and moreover this misclassification can influence the understanding and development of allergy (Fig. 1).

In the diagnosis and management of patients, diagnostic labels are important since they drive investigation and treatment strategies. The misunderstanding of diseases’ concepts is likely to hamper the indication of diagnostic method procedures as well as can induce inappropriate management. For example, using the term wheezy bronchitis to describe asthma steers people towards prescribing antibiotics for exacerbations. Some physicians and health care professionals consider wheezy bronchitis to be attributable to bacteria without appreciation that often viruses such are responsible for symptoms.

In epidemiology, terminologies and concepts are currently translated into codes and these data provide morbidity and mortality statistics. These data build a global picture and are used as the basis of health policy. If the records are unable to provide reliable data, decreasing the visibility of some conditions in detriment to others, there is a possibility of negative outcomes in health decision-making and management actions, affecting the supply and demand of goods and services at both national and global levels. As an example, research showed an under-notification of anaphylaxis deaths due to difficult coding under the ICD-10 using the Brazilian national mortality database, given that there are no anaphylaxis-specific ICD-10, which are considered valid for coding underlying causes-of-death [1].

In an economic context, these data are also able to provide aggregated indicators to understand how the whole health economy functions and, therefore, influence directly the allocation of resources for both management and research. Indeed, the weak identity of the allergy specialty in the international health classification and coding systems contributes to the lack of ascertainment and recognition of their importance for healthcare planning and resource allocation, and prevents clinical research from being performed, especially in countries in which allergy is not an academic discipline. For example, the lack of realistic anaphylaxis mortality epidemiological data to support public and private decision-making to offer appropriate treatment, such as auto-injectable adrenaline, still missing in some countries [1, 2].

In an educational context, although we now have a culture of evidence-based medicine, the dissemination of misconceptions of medical terms and definitions, even informally, can impact negatively in the formation of the new generations of health professionals. Furthermore, in today’s world, medical information is widely available through both print and online media and the source of information that patients rely on are frequently written by non-medical professionals or are based on lack of evidence data, what can perpetuate the misconceptions [3].

### Evidence for the need of reviewing the allergy and hypersensitivity definitions and the associated semantic framework

Allergy and hypersensitivity, previously perceived as simple and rare disorders, are now common and increasingly a major global public health problem. Numerous reports over the last 20 years have been indicating that the world is dealing with an allergy epidemic [4, 5]. They are complex conditions able to be expressed in many different organs and in any age, having a significant impact on the quality of life of patients and their families [6–9]. All health care professionals, in whatever role may thus encounter patients with allergic conditions.

**Fig. 2:**
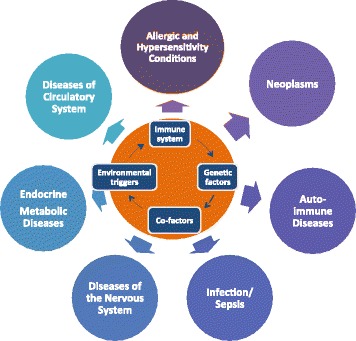
Immune system interactions and main clinical outcomes

Concepts in medicine and the new knowledge generated in the last years [4–50] have substantially changed our view of the immune system and its interaction with the environment and external agents (Fig. 2). Such developments in pathophysiology, pharmacology and clinical practice necessitate reviewing current definitions and terminologies.

The World Health Organization (WHO) decided for starting the 11th revision process with the aim of adapting the ICD frame to the new knowledge generated since the last revision. However, allergic and hypersensitivity conditions have not been adequately tracked in the ICD framework as previously demonstrated [1, 10]. Since 2013, an international collaboration of Allergy Academies, composed first by the American Academy of Allergy Asthma and Immunology (AAAAI), the European Academy of Allergy and Clinical Immunology (EAACI), the World Allergy Organization (WAO), and then by the American College of Allergy Asthma and Immunology (ACAAI), the Asia Pacific Association of Allergy, Asthma and Clinical Immunology (APAAACI) and the Latin American Society of Allergy, Asthma and Immunology (SLAAI), has worked to improve and update the classification of allergies in the forthcoming ICD-11 version, by providing scientific evidence for the need for changes [1, 10–17]. A major achievement of this process is the construction of an “Allergic and hypersensitivity conditions” parented subchapter (under the “Disorders of the immune system” chapter) guided by the World Health Organization (WHO) ICD representatives [18]. However, during the detailed technical/scientific labor-intensive building process in which we had to reach a consensus with all the “sister-specialties” with whom we have overlapping conditions, it became clear that definitions and concepts currently used in routine allergy clinical practice are not broadly known outside our community.

For all the reasons mentioned above, we have compiled this review which addresses the concepts and semantic frameworks of key allergic and hypersensitivity conditions with the idea that this should be accessible to all health care professionals in clinical practice, education and research. It is hoped that this revision will be globally accepted as a tool of improving the communication between health professionals and also support the tremendous efforts of the Joint Allergy Academies to update and standardize the allergy and hypersensitivity definitions throughout the new ICD-11.

### The strategy for a better classification of allergic and hypersensitivity diseases in the ICD-11

The 2004 European Academy of Allergy and Clinical Immunology (EAACI) - World Allergy Organization (WAO) revised nomenclature [19] was the basis of the proposed document, updated by new knowledge generated since its publication [19–50]. We also took the advances in the ICD-11 revision process to support this review manuscript since all the “allergic and hypersensitivity conditions” chapter was originally constructed by crowdsourcing the allergy community leadership [15]. Briefly, after a large survey of the allergy community [14], the identification of the gaps and trade-offs in both ICD-10 and ICD-11 beta phase codes [12] a classification model for allergic and hypersensitivity conditions has been constructed following the ICD/WHO rules and validated by crowdsourcing allergist leaderships’ community [17, 51]. The classification proposal has been presented and endorsed by the WHO revising steering group. The simplified constructed framework was the basis for the construction of the “Allergic and hypersensitivity conditions” parented subchapter into the ICD-11 beta draft [18].

Once the new section has been built, we have been moving actions to try to support awareness by disseminating updated concepts in the field. Aligned with the ICD philosophy of being of global use, this document intends to be basic, fundamental and broad to make the allergy concepts fully understood by different health professionals worldwide, besides supports the formation of in training students.

Although some countries use national modifications of ICD-10, such as United States of America (ICD-10-CM), Australia (ICD-10-AM) and Canada (ICD-CA), they may be advised by the WHO to move to the ICD-11 once it is available and proven stable. There is a substantial improvement of allergic and hypersensitivity conditions codes into the ICD-10 CM when compared with the ICD-10, even the most recent version of it [51]. However, we observe that the ICD-10 CM, as well as most of the other national adaptations, keeps the same framework, inheriting many trade-offs. The simple search of the term “Allergy” into both ICD-10 (2016 version) [51] and ICD-10 CM [52] platforms addresses to the T78 section, entitled “Adverse effects, not elsewhere classified”.

## The allergy and hypersensitivity definitions and semantic framework

### The allergy and hypersensitivity definitions

**Fig. 3:**
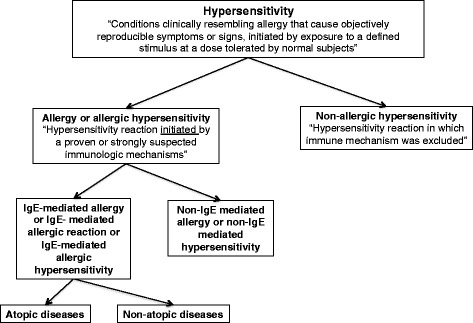
Schematical hierarchy considered for the hypersensitivity, allergy and atopy definitions, adapted from [19]

Outside the allergy community, the terms “hypersensitivity” and “allergy” have often been considered to be synonymous. However, currently in the field of allergy they receive different hierarchical positions since (Fig. 3) “*hypersensitivity*” is defined as “conditions clinically resembling allergy that cause objectively reproducible symptoms or signs, initiated by exposure to a defined stimulus at a dose tolerated by normal subjects” and “*allergy*” as “a hypersensitivity reaction initiated by proven or strongly suspected immunologic mechanisms” [19]. Therefore, the heading “hypersensitivity” includes allergic hypersensitivity conditions, such as milk-induced anaphylaxis (IgE-mediated) or antiepileptic-induced Stevens-Johnson syndrome (non-IgE mediated), as well as non-allergic hypersensitivity conditions, such as angiotensin-converting enzyme inhibitor induced angioedema or cold-induced urticaria (non-immune mediated hypersensitivity). An allergic reaction occurs when triggered by allergens to which the affected individual is sensitized (i.e., has immune antigen receptors directed against them). Although the term “intolerance” has been wrongly used to describe allergic clinical presentations by other specialties and laypersons, it has been mentioned as a non-allergic (non-immunological) hypersensitivity condition [50], out of the area of allergy practice. Therefore, this term has been added as exclusion of allergy in the new ICD-11 framework.

Table 1 shows some misunderstandings related to the concepts of “allergy”, “sensitization”, “atopy” and “atopic diseases”. The concept of “allergy” is an umbrella that covers both IgE- and non-IgE mediated conditions with different degrees of severity and is not synonymous with “atopy” or in cases of increased isolated total or specific IgE serum level. The diagnosis of allergy is based on a compatible clinical history and in vivo and/or in vitro tests to prove underlying (allergy) mechanism and etiology (cause). Therefore the tests cannot be used in isolation, for example to screen the general population for allergic conditions, because many people are sensitized, but have no allergic condition. The term atopy is used when individuals have an IgE sensitization as documented by IgE antibodies in serum or by a positive skin prick test.

**Table 1:** Current definitions for the terms “allergy”, “sensitization”, “atopy” and “atopic diseases”

Conditions	Allergy	Sensitization	Atopy	Atopic diseases
Concepts	Allergy is a hypersensitivity reaction initiated by proven or strongly suspected immunologic mechanisms. It can be IgE-mediated or non-IgE mediated. The triggers are substances that the subject has been previously exposed and sensitized.	Sensitization is considered when an underlining immune mechanism is proven by an in vivo or in vitro procedure methods, such as presence of specific IgE or T lymphocyte to an allergen. The sensitization has to be associated to a specific compatible clinical history to lead to the diagnosis of allergy.	Personal and/or familial tendency, usually in childhood or adolescence, to become sensitized and produce IgE antibodies in response to ordinary exposures to allergens, usually proteins.	Development of typical symptoms of asthma, rhinoconjunctivitis, or eczema in atopic patients. These clinical presentations can happen isolated or in combination and, in general, have a different course throughout life.

Since the concept of “hypersensitivity” covers many different conditions such as asthma, rhinitis, anaphylaxis, drug, food, and insect hypersensitivity, eczema, urticaria and angioedema, we have focused on the current definitions of conditions covered by the allergy specialty (Table 2) and to update them to the new ICD-11 “Allergic and hypersensitivity conditions” chapter. As shown in Table 2, the semantic framework on using the nomenclature “allergic” and “non-allergic” has been extended to most of the conditions. We would like to highlight the terminology used for “anaphylaxis” now used throughout the world to cover both allergic and non-allergic anaphylaxis. The term “anaphylactoid”, that was used to connote non-allergic anaphylaxis, has fallen into disuse. Another example is the term “intrinsic” or “extrinsic” used for asthma. These terms are still listed in the ICD-10 (and adaptations) and used to be in use to evoke underlying mechanism. However, following the new concepts, the terms have been tuned to “allergic asthma” and “non-allergic asthma” and now implemented into the new ICD-11.

**Table 2:** Current definitions of conditions covered by the allergy specialty and updated to the new ICD-11 “Allergic and hypersensitivity conditions” chapter

Main groups of allergic and hypersensitivity conditions [15, 19]	Definitions for allergic and hypersensitivity conditions implemented the ICD-11 beta draft platform	Corresponding subchapter into the new “Allergic and hypersensitivity conditions” ICD-11 chapter (ICD-11 beta draft Foundation September 2015 version) [18]
Rhinitis	Rhinitis is an inflammation of the nasal mucosa clinically characterized by major symptoms: sneezing, nasal pruritus, running nose, and stuffy nose.	Allergic and hypersensitivity disorders involving the respiratory tract
Allergic rhinitis	Allergic rhinitis is an inflammation of nasal airway triggered by allergens to which the affected individual has previously been sensitized. Pathogenesis of allergic rhinitis is type I IgE-mediated allergy on the nasal mucosa. Antigens inhaled into sensitized nasal mucosa bind to IgE antibodies on mast cells, which release chemical mediators such as histamine and leukotrienes. The main triggers are inhaled allergens, such as house dust mites and pollens.	
Non-allergic rhinitis	Non-allergic rhinitis is an inflammation of nasal mucosa in which allergic mechanisms are not involved. It covers many different phenotypes and the major symptoms (sneezing, running nose, and stuffy nose) with variable intensity according to the triggers/causes.	
Asthma	Asthma is a clinical syndrome characterized by recurrent attacks of breathlessness and wheezing or cough, which vary in severity and frequency from person to person. In an individual, they may occur from hour to hour and day to day. Allergic and non-allergic asthma are a heterogeneous group of disorders due to inflammation of the air passages in the lungs and affects the sensitivity of the nerve endings in the airways so they become easily irritated.	
Allergic asthma	Allergic asthma is the most easily recognized asthma phenotype, which often commences in childhood and is associated with a past and/or family history of allergic disease such as eczema, allergic rhinitis, or food or drug allergy. Examination of the induced sputum of these patients before treatment often reveals eosinophilic airway inflammation. The main triggers are inhaled allergens, such as house dust mites and pollens. Patients with this asthma phenotype usually respond well to inhaled corticosteroid (ICS) treatment depending on the severity.	
Non-allergic asthma	Non-allergic asthma occurs in some patients who have asthma that is not associated with allergy. The cellular profile of the sputum of these patients may be neutrophilic, eosinophilic or contain only a few inflammatory cells (paucigranulocytic). Patients with non-allergic asthma often respond less well to ICS. It can cover different phenotypes, such as Aspirin induced asthma, virus induced asthma, exercise induced bronchospasm.	
Conjunctivitis	Conjunctivitis is the inflammation of the conjunctiva. It can have many different causes and can cover both allergic and non-allergic conjunctivitis. Allergic conjunctivitis is an IgE-mediated response due to the exposure of seasonal or perennial allergens in sensitized patients. The allergen-induced inflammatory response of the conjunctiva results in the release of histamine and other mediators. Symptoms consist of redness (mainly due to vasodilation of the peripheral small blood vessels), edema (swelling) of the conjunctiva, itching, and increased lacrimation (production of tears). Besides IgE-mediated conjunctivitis, contact allergic conjunctivitis involving TH1 mechanisms also occurs.	Allergic or hypersensitivity disorders involving the eye
Dermatitis	Local inflammation of the skin, that can cover both immune-mediated and non-immune mediated conditions.	Allergic or hypersensitivity disorders involving skin and mucous membranes
Contact dermatitis	*Allergic contact dermatitis* is an eczematous response provoked by a type IV delayed immune reaction in the skin to a substance or substances to which the individual has previously been sensitized. *Non*-*allergic contact dermatitis* is usually due to external irritants; it is an eczematous reaction provoked by acute or prolonged and repeated contact with a substance or substances which are injurious to the skin. Common irritants include defatting agents (solvents, soaps and detergents), acids (both inorganic and organic) and alkalis (e.g., sodium hydroxide and wet cement).	
Atopic eczema	A chronic inflammatory genetically determined eczematous dermatosis associated with an atopic diathesis (elevated circulating IgE levels, Type I allergy, asthma and allergic rhinitis). It is manifested by intense pruritus, exudation, crusting, excoriation and lichenification. Often presenting in infancy affecting the face, forearms and lower limbs, it tends to move to the limb flexures after infancy. Although commonly limited in extent and duration, it may be generalized and life-long.	
Urticaria (or Spontaneous urticaria)	Urticaria is a disease characterized by the development of wheals (hives), angioedema, or both. It is classified as acute when it lasts less than six weeks, and chronic when lasts six weeks or more. When the reaction is mediated by immunological mechanisms, the term should be allergic urticaria.	
Anaphylaxis	Anaphylaxis is a severe, life-threatening systemic hypersensitivity reaction which is rapid in onset with potentially life-threatening airway, breathing, or circulatory problems and is usually, although not always, associated with skin and mucosal changes. It can be allergic or non-allergic.	Anaphylaxis
Food hypersensitivity	Food hypersensitivity reactions are adverse effects of food or food additives that clinically resemble allergy. Food allergy is an adverse reaction to food mediated by an immunologic mechanism, involving specific IgE (IgE-mediated), cell-mediated mechanisms (non-IgE-mediated) or both IgE- and cell-mediated mechanisms (mixed IgE- and non-IgE-mediated).	Complex allergic or hypersensitivity conditions
Drug hypersensitivity	Drug hypersensitivity reactions are the adverse effects of pharmaceutical formulations (including active drugs and excipients) that clinically resemble allergy. It belongs to type B adverse drug reactions, which are defined by the World Health Organization as the dose-independent, unpredictable, noxious, and unintended response to a drug taken at a dose normally used in humans. It covers many different clinical phenotypes with variable onset and severity.	
Hymenoptera and other insects hypersensitivity or allergy	Hymenoptera and other insects’ hypersensitivity cover local cutaneous reactions (large local reactions) and anaphylaxis due to contact to the venom (sting, bite) or saliva (bite) of insects (e.g., bee, wasp, tick). These reactions can be immune mediated (e.g., IgE-mediated or non-IgE-mediated venom allergy) or non-immune mediated.	

The development of medicine and new technological knowledge has dramatically changed the landscape in which we practice medicine. Within the specialty of clinical allergy, many diagnostic procedures have emerged to support the correct diagnosis of allergic and hypersensitivity conditions [48] (Table 3). However, apart from minor modifications, the in vivo tests, such as skin prick and patch tests, still resemble the original methods described. In this regard, we would like to further underline that skin tests and in vitro procedures (Table 3) are not usually indicated as a screening of the general population (without symptoms and a working or suspected diagnosis). The tests are used to support diagnosis in patients with a suspicious history and have to be carefully interpreted. The management of allergy and hypersensitivity has also been affected with the implementation of novel therapeutic agents, drug classes and new devices to optimize the best treatment of these patients. For this reason, the concepts used for desensitization/tolerance induction and immunotherapy have been recently reviewed. From the allergy specialty perspective, all allergen immunotherapies (AIT) are desensitization procedures. However, the desensitization/tolerance induction procedures can be used with allergens (procedure named AIT) or other products not made of allergens (e.g., AIT for allergic rhinitis), procedure named allergy immunotherapy (e.g., the anti-IgE monoclonal antibody omalizumab for asthma). It can address both allergic hypersensitivity conditions, such as IgE-mediated food-induced anaphylaxis (e.g., milk-induced anaphylaxis), as well as non-immune-mediated hypersensitivity conditions (e.g., NSAIDs-exacerbated respiratory disease) [49].

**Table 3:** Main current diagnostic procedures for allergy/hypersensitivity (adapted from [48])

IgE-mediated hypersensitivity diagnostic procedures	T-Lymphocyte-mediated hypersensitivity diagnostic procedures
In vitro − Serum allergen specific-IgE − Serum (or plasma) tryptase − Basophil activation test (BAT) − Cellular Antigen Stimulation Test (CAST)- Enzyme linked ImmunoSorbent Assay (ELISA)	In vitro − Lymphocyte transformation blood test − Enzyme-Linked ImmunoSpot (ELISPOT) − CD69 expression
In vivo − Skin tests • Skin prick tests • Intradermal skin tests − Provocation test	In vivo − Skin tests • Skin patch tests/Photopatch tests • Intradermal skin tests − Provocation test

## Conclusion

### The allergic and hypersensitivity in process

**Fig. 4:**
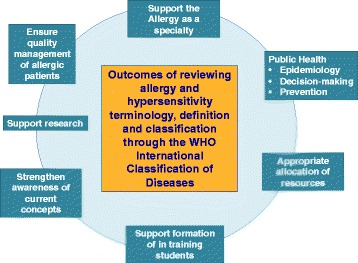
Expected positive outcomes of reviewing allergy and hypersensitivity terminology, definition and classification

The allergic and hypersensitivity concepts have been evolving substantially and efforts have been addressed to allow harmonization of work definitions and terminology. The classification systems are used to categorize ideas and concepts to support the real-life management decision-making and are, in general, translated automatically into codes. In clinical practice, clinicians select diagnostic labels based on classification knowledge. This current review intends to be accepted and used universally by all health professionals involved in diseases’ classification and coding and, therefore, contribute to improve care and outcomes in this increasing sub-section of the world’s population (Fig. 4).

## Abbreviations

AAAAI, American Academy of Allergy Asthma and Immunology; ACAAI, American College of Allergy, Asthma and Immunology; AIT, allergen immunotherapies; APAAACI, Asia Pacific Association of Allergy, Asthma and Clinical Immunology; BAT, basophil activation test; CAST, Cellular Antigen Stimulation Test; EAACI, European Academy of Allergy and Clinical Immunology; ELISPOT, Enzyme-Linked ImmunoSpot; ICD, International Classification of Diseases; IgE, immunoglobulin E; SLAAI, Latin American Society of Allergy, Asthma and Immunology; WAO, World Allergy Organization; WHO, World Health Organization
